# Surgical and molecular pathology of pancreatic neoplasms

**DOI:** 10.1186/s13000-016-0497-z

**Published:** 2016-06-07

**Authors:** Wenzel M. Hackeng, Ralph H. Hruban, G. Johan A. Offerhaus, Lodewijk A. A. Brosens

**Affiliations:** Department of Pathology, University Medical Center Utrecht, Heidelberglaan 100, 3584 CX Utrecht, The Netherlands; Department of Pathology, The Sol Goldman Pancreatic Cancer Research Center, The Johns Hopkins University School of Medicine, Baltimore, MD USA

**Keywords:** Pancreas, Pancreatic cancer, Acinar cell carcinoma, Pancreatic neuroendocrine tumor, Solid-pseudopapillary neoplasm, Genetics, Histology, Methylation, microRNA, Sequencing

## Abstract

**Background:**

Histologic characteristics have proven to be very useful for classifying different types of tumors of the pancreas. As a result, the major tumor types in the pancreas have long been classified based on their microscopic appearance.

**Main body:**

Recent advances in whole exome sequencing, gene expression profiling, and knowledge of tumorigenic pathways have deepened our understanding of the underlying biology of pancreatic neoplasia. These advances have not only confirmed the traditional histologic classification system, but also opened new doors to early diagnosis and targeted treatment.

**Conclusion:**

This review discusses the histopathology, genetic and epigenetic alterations and potential treatment targets of the five major malignant pancreatic tumors - pancreatic ductal adenocarcinoma, pancreatic neuroendocrine tumor, solid-pseudopapillary neoplasm, acinar cell carcinoma and pancreatoblastoma.

## Background

Malignant neoplasms of the pancreas are currently classified based on the cellular direction of differentiation (ductal, acinar or neuroendocrine) of the neoplastic cells, combined with the macroscopic appearance (solid or cystic) of the tumors. Pancreatic ductal adenocarcinoma comprises about 90 % of all malignant pancreatic neoplasms. Of all other malignant pancreatic neoplasms (pancreatic neuroendocrine tumors, solid-pseudopapillary neoplasm, acinar cell carcinoma and pancreatoblastoma), neuroendocrine tumors are the most common, comprising approximately 5 % of malignant pancreatic tumors (Table 1).

**Table 1:** Differential diagnosis of malignant pancreatic neoplasms. Overview of pancreatic neoplasms with their relative prevalence, direction of differentiation, macroscopic and microscopic appearance, and immunohistochemical markers

	Prevalence (% of all malignant pancreatic tumors)	Mean Age (SD) in years	Sex predominance	Direction of differentiation Ductal/acinar/endocrine	Gross: Solid/solid and cystic/cystic	Microscopic	Immunohistochemical
Pancreatic ductal adenocarcinoma	90 %	66 (11)	Male (3:2)	Ductal	Solid	• Glandular and ductal structures • Abundant desmoplastic stroma • Eosinophilic to clear cytoplasm and enlarged pleomorphic nuclei • Perineural, lymphatic and blood vessel invasion	Aberrant TP53 expression, SMAD4 loss, expression of MUC1, MUC3, MUC4, MUC5AC, CA19-9
Pancreatic neuroendocrine tumor/carcinoma	5 %	58 (15)	Male (3:2)	Endocrine	Solid, sometimes cystic degeneration	• Nested or trabecular growth pattern • Granular amphophilic to eosinophilic cytoplasm • “Salt and pepper” chromatin	Expression of synaptophysin and chromogranin, peptide hormones (e.g. insulin and glucagon), aberrant nuclear TP53 expression in PanNECs
Solid-pseudopapillary neoplasm	1–2 %	29 (14)	Female (9:1)	Uncertain	Solid and cystic	• Poorly cohesive uniform cells • Extensive degenerative changes. • Eosinophilic or clear vacuolated cytoplasm Round to oval nuclei, often grooved or indented. • Eosinophilic globules and foamy macrophages	Abnormal nuclear labeling for β-catenin, expression of CD10, paranuclear dot-like CD99 labeling or lymphoid enhancer-binding factor 1 (LEF1). Loss of membranous E-cadherin
Acinar cell Carcinoma	1–2 %	56 (15) 6 % between 8 and 15	Male (2:1)	Acinar	Solid, sometimes cystic degeneration	• Enlarged uniform nuclei with prominent nucleoli • Finely granular eosinophilic cytoplasm. • Small acinar units or sheets	BCL10, expression of pancreatic exocrine enzymes: trypsin, chymotrypsin, lipase
Pancreatoblastoma	<1 %	5 (2), second peak around 40	Slightly male	Acinar	Solid, cystic in BWS ^a^	• Similar to ACC • Squamoid nests required for diagnosis • Neuroendocrine or ductal component.	Expression of pancreatic exocrine enzymes, BCL10, SMAD4 loss, Abnormal nuclear labeling for β-catenin

^a^ *BWS* Beckwith-Wiedemann syndrome

Recent genetic and epigenetic characterization of these histologically distinct pancreatic tumors has increased our understanding of common genetic signatures, and has also identified tumor specific genetic alterations (Table 2). In addition to serving as diagnostic tools, some genetic alterations can be exploited as targets for therapy, opening avenues for new treatments. In this review, histology, genetics and epigenetics of malignant pancreatic tumors and potential targets for treatment are discussed.

**Table 2:** Overview of pancreatic neoplasms with their key genetic alterations and several epigenetic alterations discussed in this review

	Average number of somatic mutations	Major genes involved	Methylation	MiRNA tumor expression compared to normal pancreatic tissue
Pancreatic ductal adenocarcinoma	20–80	*KRAS*, *CDNK2A*, *SMAD4*, *TP53*, *MLL3*, *TGFBR2*, *ATM*, *ARID1A*, *ROBO2*, *KDM6A*	Loss of function through promotor hypermethylation: *CDNK2A*, *hMLH1*,	Upregulation: miR-21, 23a, 31, 100, 143, 155, and 221
				Downregulation: miR-148a, 217 and 375
Pancreatic Neuroendocrine tumor/carcinoma	16	*MEN1*, *ATRX*, *DAXX*, *TSC2*, *PTEN* ^#^, *Rb*, TP53*	Hypomethylation of *LINE1* and hypermethylation of *RASSF1A* promoting the accumulation of β-catenin	Upregulation: miR-193b, 103 and 107
				Downregulation: miR-155
Solid-pseudopapillary neoplasm	3	*CTNNB1*	*u*	MiRNAs possibly upregulating the Wnt, Hedgehog, and Androgen receptor pathway
Acinar cell carcinoma	131	*SMAD4*, *JAK1*, *BRAF*, *RB1*, *TP53*, *APC*, *ARID1A*, *GNAS*, *MLL3*, *PTEN*	Hypermethylation of *RASSF1*, *MLH1* and *APC*	Upregulation: miR-17, 20, 21, 92–1, 103, 107
				Downregulation: miR-155
Pancreatoblastoma	18	Loss of chromosome 11p, *CTNNB1*	Hypermethylation of *RASSF1A*	u

*u* unknown. ^#^ *MEN1*, *ATRX*, *DAXX*, *TSC2* and *PTEN* mutations are found in well-differentiated PanNET but not in PanNEC. * *Rb* and *TP53* mutations are present in PanNEC, but not in well-differentiated PanNET

### Pancreatic ductal adenocarcinoma

Infiltrating ductal adenocarcinoma, also known as pancreatic ductal adenocarcinoma (PDAC), accounts for 90 % of all malignant pancreatic neoplasms and occurs at a mean age of 66 years [1]. PDAC has a very poor prognosis with an overall 5-year survival of only 7 % [2]. At diagnosis, the majority of patients are inoperable due to locally advanced or metastatic disease. The median survival for patients with metastatic disease is less than a year [3]. Moreover, by the year 2030 pancreatic cancer is predicted to become the second leading cause of cancer-related death in the U.S. [4]. In view of the increasing incidence and the virtually unchanged poor prognosis of PDAC both new therapies for established pancreatic cancer as well as methods for prevention and early detection are desperately needed.

#### Gross and microscopic findings

**Fig. 1:**
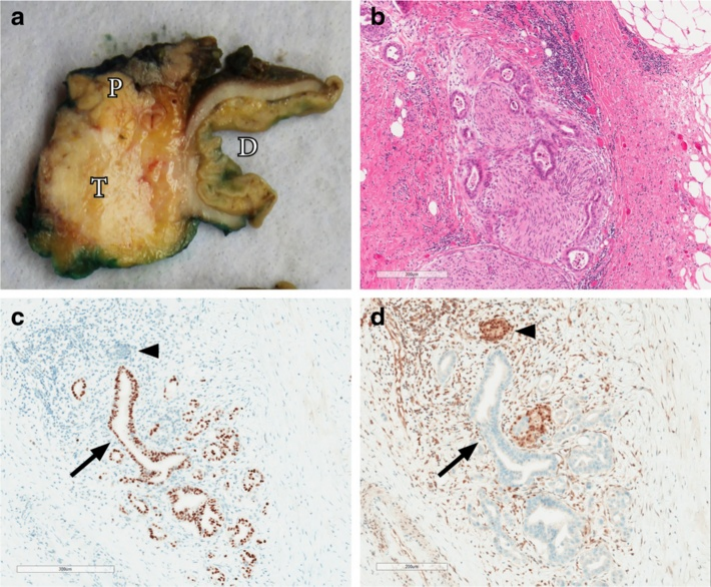
**a** Macroscopic appearance of a pancreatic ductal adenocarcinoma showing a poorly demarcated firm white tumor in the pancreatic parenchyma (*T* Tumor, *P* pancreatic parenchyma, *D* duodenum). **b** Perineural invasion of a pancreatic ductal adenocarcinoma. **c** Positive TP53 immunohistochemistry in pancreatic ductal adenocarcinoma indicative of *TP53* gene mutation. *Arrow*, malignant ductal structure; *arrowhead*, normal pancreatic duct. **d** Loss of SMAD4 immunohistochemistry in pancreatic ductal adenocarcinoma indicating mutation of the *SMAD4* gene. *Arrow*, malignant ductal structure; *arrowhead*, normal pancreatic duct

PDACs are characteristically firm, ill-defined white-yellow masses (Fig. 1a). The pancreatic parenchyma upstream from PDACs is usually atrophic and the main pancreatic duct can be dilated. Microscopically, PDAC is composed of haphazardly arranged infiltrating glandular and ductal structures typically surrounded by abundant desmoplastic stroma. The cells have eosinophilic to clear cytoplasm and usually enlarged pleomorphic nuclei. Poorly differentiated ductal adenocarcinomas have more irregular and smaller glands and significant pleomorphism. Perineural, lymphatic and blood vessel invasion are frequently present (Fig. 1b). The neoplastic cells in areas of venous invasion can be so well-differentiated that they mimic non-invasive precursor lesions (pancreatic intraepithelial neoplasia). Immunohistochemically, there is no definite marker to distinguish PDAC from non-neoplastic ductal structures, although aberrant TP53 expression or SMAD4 loss support the diagnosis of PDAC over reactive glands (Fig. 1c and d) [5, 6]. Several types of mucin (MUC1, MUC3, MUC4, MUC5AC) and glycoprotein tumor antigens such as CA19-9 can be expressed in PDAC [7–9]. The main microscopic differential diagnosis consists of PDAC precursor lesions, other malignant pancreatic tumors (Table 1), pancreatitis and adenocarcinoma metastasis.

**Fig. 2:**
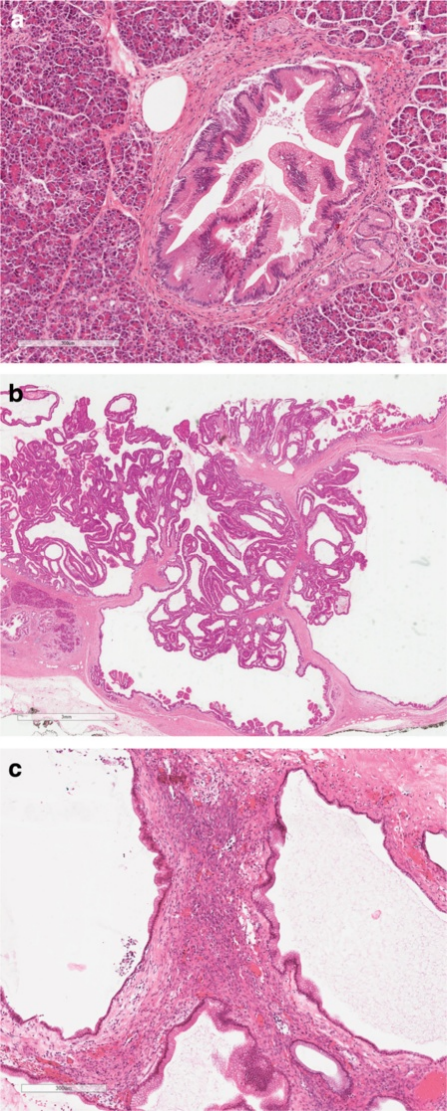
**a** Low-grade pancreatic intraepithelial neoplasia (PanIN) showing micro-papillary epithelium with mild to moderate cytological atypia. **b** Intraductal papillary mucinous neoplasm (IPMN), gastric-foveolar type with low-grade dysplasia. **c** Mucinous cystic neoplasm (MCN) showing gastric-foveolar type epithelium with low-grade dysplasia, surrounded by ovarian-type stroma

**Fig. 3:**
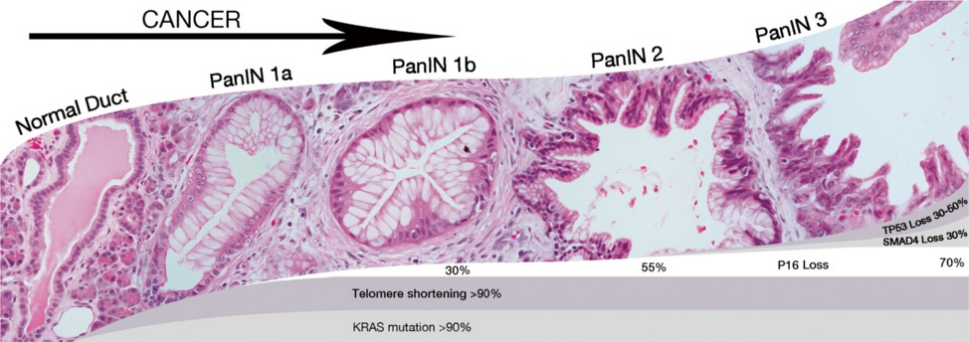
Pancreatic cancer develops from the well-defined precursor lesions pancreatic intraepithelial neoplasia, intraductal papillary mucinous neoplasm and mucinous cystic neoplasm. The PanIN progression model shown here shows that accumulation of genetic and epigenetic alterations drives neoplastic progression in these precursor lesions from low-grade dysplasia (PanIN 1 and PanIN 2) to high-grade dysplasia (PanIN 3) to eventually an invasive pancreatic adenocarcinoma

PDAC develops from precursor lesions that can be either microscopic (pancreatic intraductal neoplasia, PanIN) or macroscopic cystic precursor lesions (intraductal papillary mucinous neoplasm, IPMN; mucinous cystic neoplasm, MCN) (Fig. 2). IPMN and MCN are often found as incidental finding on imaging. PanIN arises in microscopic ducts; IPMN arises within the main- or branch-ducts. MCN usually does not communicate with the ductal system. Microscopically, all precursors show flat or papillary mucin-producing neoplastic epithelium, with varying degrees of dysplasia and directions of differentiation. Stepwise accumulation of (epi)genetic alterations drives neoplastic progression and eventually development of malignant invasive carcinoma, analogous to the PanIN progression model as depicted in Fig. 3 and discussed below [10, 11].

#### Genetic signature: familial PDAC

Approximately 10 % of pancreatic cancers appear to have an inherited component. Overall, sporadic and familial PDAC share the same driver mutations (*KRAS*, *CDKN2A*, *TP53* and *SMAD4*) [12], but some of these cases are caused by inherited germline genetic alterations in genes that significantly increase the risk of pancreatic cancer (Table 3). These genes include *BRCA2*, *BRCA1*, *PALB2*, *p16*/*CDKN2A*, *ATM*, *STK11*, *PRSS1*, and the DNA mismatch repair genes (such as *MLH1* and *MSH2*) [13–17]. In addition, a number of other candidate genes, such as *BUB1B*, *CPA1*, *FANCC* and *FANCG*, have been described [12]. These germline alterations are critical to understand because the risk is significant and at-risk patients can be enrolled in screening and early detection protocols for pancreatic and extra-pancreatic tumors [18]. In addition, some patients with specific genetic alterations can be prioritized for specific therapies. For example, some tumors characterized by microsatellite instability due to a DNA mismatch repair gene defect are exquisitely responsive to immunotherapies, and some tumors with *BRCA* or *PALB2* gene mutation are sensitive to poly ADP ribose polymerase (PARP)-inhibitors [19–21].

**Table 3:** Overview of germline genetic alterations with well-defined pancreatic cancer risk and genes that have been associated with familial PDAC

Gene (syndrome)	RR (Cumulative lifetime risk (%) by age 70)
*STK11*/*LKB1* (Peutz-Jeghers syndrome)	132 (36)
*PRSS1*/*SPINK1* (hereditary pancreatitis)	50–80 (40)
*CDKN2A* (FAMMM)	13–47 (17)
*BRCA1*/*BRCA2* (HBOC)	3.5–10 (3–8)
*MLH1*, *MSH2*, *MSH6*, *PMS2* (*Lynch syndrome*)	8.6 (<5)
*CFTR* (cystic fibrosis)	5 (<5)
FDR with PC	2–3 (2)
FDRs with PC	6 (8–12)
Possible role in FPC: *ATM*, *TET2*, *DNMT3A*, *POLN*, *POLQ*, *ASXL1*, *PALB2*, *FANCG*, *BUB1B*, *ESCO2*, *FANCC*, *FANCM*, *MSH4*, *RAD54L*	Unknown

*RR* relative risk, *FDR* first degree relative, *FAMMM* familial atypical multiple mole melanoma, *HBOC* hereditary breast and ovarian cancer syndrome, *FAP* familial adenomatous polyposis, *PC* pancreatic cancer, *FPC* familial pancreatic cancer. Adapted from Ghiorzo et al. and Roberts et al. [12, 151]

In addition to these low prevalence but high penetrance genes, there are a number of more common lower penetrance genes that increase the risk of pancreatic cancer only slightly. A number of these, including ABO blood group type, have been identified in genome wide association studies (GWAS) [22–24].

#### Genetic signature: sporadic PDAC

The somatic alterations present in PDAC are now well characterized thanks to several large whole-exome and whole-genome sequencing studies [21, 25–27]. On average PDACs have 50–80 exomic non-silent mutations [21, 25–27]. In addition, extensive larger structural variations including intra-chromosomal rearrangements, deletions and amplifications are common in PDAC [21, 28].

Point mutation of the oncogene *KRAS* is seen in almost all early pancreatic cancer precursor lesions and in PDACs. Subsequent mutations that drive neoplastic progression in PanIN lesions are usually in the tumor suppressor genes *CDKN2A*, *TP53* and *SMAD4* (Fig. 3) [21, 25, 26]. Further accumulation of genetic and epigenetic alterations drives neoplastic progression in these precursor lesions, eventually leading to an invasive pancreatic adenocarcinoma [10]. Less commonly mutated genes in PDAC include *MLL3*, *TGFBR2*, *ATM*, *ARID1A*, *ROBO2* and *KDM6A* [21, 25–27]. Of note, mutations in chromatin-regulating genes (*MLL*, *MLL2*, *MLL3* and *ARID1A*) have been associated with improved survival, and loss of *SMAD4* with poorer survival [29, 30]. Many mutations found by whole exome sequencing are reported in a very low percentage of tumors, and therefor categorized as passengers in tumorigenesis. Of note the recently proposed “mini driver” model hypothesizes that several passengers might have relatively weak tumor-promoting effects but together might substitute for a major-driver [31]. More research is needed to address the exact role of most of these less prevalent mutations in tumorigenesis.

Importantly - despite the diversity of genes targeted - the genetic alterations in PDAC appear to selectively target core signaling pathways, including Wnt/Notch signaling, TGF-β signaling, and DNA damage control [26]. Despite the genetic heterogeneity of PDAC, targeting one or more of these pathways may be more effective than targeting a specific genetic alteration. For example, Waddell et al. recently correlated deleterious mutations in *BRCA1* and *BRCA2* with unstable genetic signatures (>200 structural variations). In their study, 4 out of 5 patients with defective DNA damage control responded to treatment with a platinum containing regimen. Also PARP inhibitors have been reported to be effective in *BRCA* mutated tumors [21]. These findings illustrate how knowledge of rare mutations in known pathways can be used to guide treatment. A number of clinical trials targeting specific pathways and mutations are being conducted on patients with PDAC and other human cancers. Potential targets for therapeutic intervention are seen in over a third of PDACs (up to 97 % when trials related to *KRAS* and *TP53* are included) [29]. Future personalized treatment might thus drastically change outcome of this disease.

Studies of the clonal evolution of genetic changes in pancreatic cancer and metastases by Yachida et al. suggest that it takes almost 12 years from the initiating mutation in the pancreas until development of an invasive PDAC [32]. This suggests a wide window of opportunity for the early detection of pancreatic cancer. The genetic alterations present in pancreatic cancer and its non-invasive precursors can be shed into the blood and into the pancreatic duct system. This suggests the possibility of gene-based early detection tests. Indeed, mutant *KRAS* shed from invasive pancreatic cancer can be detected in the plasma, and mutations present in non-invasive cystic precursor lesions, such as IPMNs and MCNs can be detected in cyst fluid aspirated at the time of endoscopic ultrasound (EUS), as well as in secretin stimulated pancreatic juice collected from the duodenum [33, 34].

#### Epigenetic alterations

A number of genes are aberrantly methylated in pancreatic cancer [35–41]. For example, integrated methylation and gene-expression meta-analysis have identified a number of genes (*MUC4*, *SERPINB5*, *CLDN4*, *SFN*, *TFF1*, *S100P*, *S100A4*, *MMP1*, *MMP7*, *MSLN*, *PSCA*, *ID1*, *MST1R*, *NBL1*, *PHLDA2*, *PLAT*, *PLAUR*, *IL8*, *SPP1*, *ARHGDIB*, *NQO1*, and *ITGB4*) that are significantly upregulated in PDAC, likely caused by promoter hypomethylation [36, 42, 43]. Some of the genes targeted by changes in methylation are clearly cancer-causing, such as the well-known tumor suppressor gene *CDNK2A* and the DNA repair gene *hMLH1*, which show loss of function through promoter hypermethylation silencing [40, 44–46].

These epigenetic changes are not only functionally important, but can also be used as markers of disease and early detection. For example, DNA methylation alterations in the pancreatic juice are a possible approach to the diagnosis of pancreatic cancer [47].

#### MicroRNA

Post-transcriptional regulation or silencing of gene expression occurs mostly by non-coding RNAs. The most studied non-coding RNAs are microRNAs (miRs), which are small single stranded RNA molecules that regulate mRNA by full or partial complementarity. Deregulated miRs can give information on transcriptional regulation and may serve as biomarkers for survival and early detection [48–50].

Recently a large meta-analysis looked at the miR expression profiles of 538 PDACs. A statistically significant miR meta-signature with upregulation of miR-21, 23a, 31, 100, 143, 155, and 221 and downregulation of miR-148a, 217 and 375 was found in PDAC. Furthermore, in a cohort of 70 patients, the high expression of miR-21, miR-31 and the low expression of miR-375 in their PDACs was found to be an independent prognostic marker for poor overall-survival [50]. Interestingly, in stool from patients with PDAC, significantly higher miR-21 and miR-155 and lower miR-216 levels have been found compared to normal controls [51]. Other studies with “disease free survival” and “overall survival” as outcome measures also found an important role for high levels of miR-21 in predicting prognosis, along with high miR-155, high miR-203, and low miR-34a [49].

MiR-21 is thus an important candidate for diagnostic and prognostic purposes, although it cannot be used to differentiate between PDAC and precursor lesions such as intraductal papillary mucinous neoplasms (IPMN) or malignancy in other organs [52, 53]. MiR-21 has approximately 180 target messenger RNAs (mRNA) [54]. Interestingly several of these targets are tumor suppressors and negative regulators of the Ras/MEK/ERK pathway. An in vivo study with a murine non-small cell lung carcinoma model confirmed upregulation of miR-21 with RAS activation, and downregulation of several negative RAS regulators and tumor suppressors including *SPRY1*, *SPRY2*, *BTG2*, and *PDCD4* [54]. In vitro studies have reported several other miR-21 affected tumor suppressor mRNAs, including PTEN [55]. Deletion of miR-21 has also been shown to repress tumor formation in *KRAS* mutated mice and makes in vitro cells more sensitive for chemotherapy, possibly by repression of the AKT pathway through p85α inhibition [56]. MiR-21 may thus be potentially interesting as pharmacological target as well.

Research on miRs is booming, and many recent studies have found other and new miRs not reported in the meta-reviews, also to be excellent prognostic markers for PDAC [57–60]. Other forms of non-coding RNA like long non-coding RNA (lncRNA), small nucleolar-derived RNA (sdRNA) and piwi-interacting RNA (piRNA) are also differentially altered in PDAC [61].

#### Changes in gene expression

Several studies have tried to classify PDAC into clinically meaningful subgroups based on gene expression profiles. Collisson et al. clustered 3 distinct subtypes of PDAC (classical, quasimesenchymal and exocrine-like) with different responses to treatment and different patient prognosis [62]. Recently Moffitt et al. used blinded digital separation of PDAC gene expression microarray data to cluster primary carcinoma, metastasis, and normal samples [63]. They found that the groups described by Collisson et al. did not hold predictive power in their samples. Instead they identified two tumor subtypes: “classical” which had great overlap with the classical group of Collison et al., and basal-like which had a worse outcome and was molecularly similar to basal tumors in the bladder. Furthermore, as they could digitally separate tumor and stromal expression, they defined “normal” and “activated” stromal subtypes, which they reported to be independent prognostic factors [63]. Currently, there is no well-established clinically meaningful subclassification of PDAC.

Differentially upregulated genes by mRNA can result in upregulation of proteins, which - just like DNA, miRNA and mRNA - can be used as potential diagnostic biomarkers of malignancy in pancreatic juice and blood [64]. Furthermore, specific mutated proteins such as mutant Ras can be distinguished from wild-type Ras by mass spectrometry in tissue and pancreatic juice, which might be even more useful for early diagnosis of PDAC and its precursors [65]. Other highly expressed proteins including mesothelin are potentially targetable with immunotherapy [66]. Mutant proteins can also give rise to aberrant epitopes on tumor MHC receptors, which then can specifically be targeted by adoptive T-cell therapy as elegantly demonstrated in other human cancers [67].

#### Stroma and the tumor microenvironment

In addition to genetic alterations, the tumor microenvironment and changes in epigenetic regulation play important roles in promoting or suppressing PDAC growth [68, 69] and stromal expression profile has shown prognostic significance [63]. Also, by overexpression of hyaluronic acid and collagens, the extracellular matrix can cause a high interstitial fluid pressure, causing compression of blood vessels and therefor hindering passive transport processes of chemotherapeutics. Targeting these stromal factors might improve therapeutic response [70].

PDAC and its microenvironment are also marked by distinct immune cell populations along its path of tumorigenesis, creating an immunosuppressive environment that shields tumor cells from detection and renders them resistant to immune-based therapies. Regulatory T-cells (T-reg) seem to play a role from the earliest stage of precursor disease potentially undermining anti-tumor effector T-cell activity; high intratumor T-reg/CD4^+^ T-cell ratio is a prognostic factor for worse survival. Therapeutically targeting of T-regs in malignancy is currently under investigation [70].

### Pancreatic neuroendocrine tumors

Pancreatic neuroendocrine tumors (PanNET) are the second most common malignant tumor of the pancreas [6]. PanNETs occur mostly in elderly patients, with a mean age of 58 years [71]. Although prognosis of PanNET is better than PDAC, it is still poor with an average overall 5 year survival of only 42 % [72]. Functional PanNETs are well known for their classic clinical presentations including Whipple’s triad (insulinoma) and Zollinger-Ellison syndrome (gastrinoma), in which hypersecretion of pancreatic or non-pancreatic hormones have systemic effects. When no systemic effects of hormone production are seen, PanNETs are by definition classified as non-functional [5].

A number of TNM classification systems with prognostic value for PanNET patients have been developed by the WHO2010 [World Health Organization], ENETs [European Neuroendocrine Tumor Society] and AJCC [American Joint Committee on Cancer] [73]. Although it is at present not completely established which system should be preferred, a recent study suggests that the ENETs TNM classification was superior to the AJCC/WHO2010 classification/grading System and more accurate [74].

#### Gross and microscopic findings

**Fig. 4:**
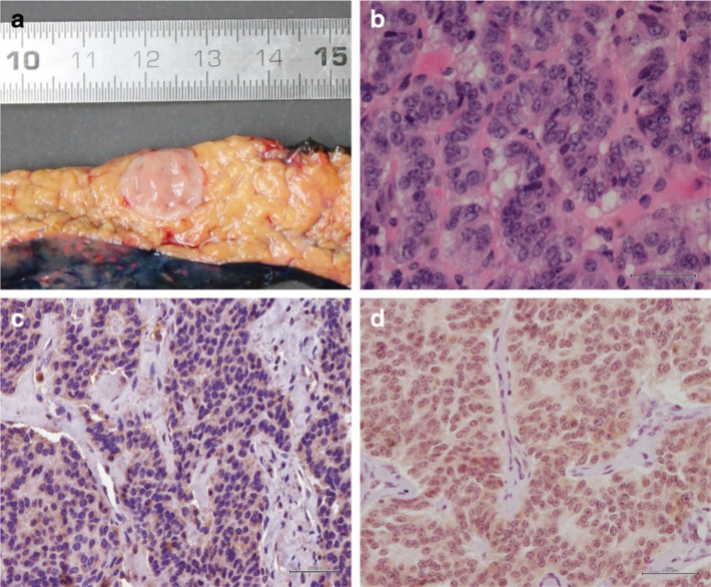
**a** Macroscopic appearance of pancreatic neuroendocrine tumor showing a well-demarcated pinkish tumor surrounded by normal pancreatic parenchyma. **b** Pancreatic neuroendocrine tumor, detail showing typical salt and pepper chromatin. **c** Loss of Menin expression in pancreatic neuroendocrine tumor indicative of *MEN1* gene inactivation. **d** Retained Menin expression in pancreatic neuroendocrine tumor with a wildtype *MEN1* gene

PanNETs are usually soft, sometimes red or white, well-demarcated lesions (Fig. 4a). Microscopically the neoplastic cells have a nested or trabecular growth pattern. At higher magnification, the neoplastic cells have a distinct neuroendocrine morphology, with a granular amphophilic to eosinophilic cytoplasm and the typical coarsely clumped “salt and pepper” chromatin (Fig. 4b). The mitotic rate and percentage of Ki67 positive cells are used for grading. The well-differentiated PanNETs can be either grade 1 (<2 mitoses per 10 HPF; Ki-67 labeling index <2 %) or grade 2, (2–20 mitoses per 10 HPF; Ki-67 labeling index 3–20 %). If mitotic count is >20 mitoses per 10 HPF or Ki-67 index is >20 %, the neoplasm is classified as a grade 3 neuroendocrine tumor or neuroendocrine carcinoma (PanNEC). Histologically PanNECs can have one of two appearances. Those with a Ki-67 <50 % can look similar to the well-differentiated PanNETs, only they have a high proliferation rate [75]. This group is somewhat more aggressive than grade 2 PanNETs but not as rapidly progressive as the PanNECs with a Ki67 >50 %. PanNECs with a very high proliferation rate (>50 %) can have a small cell carcinoma or large cell carcinoma appearance with markedly pleomorphic typical neuroendocrine cells that are tightly packed in nests or form diffuse irregular sheets [5]. Necrosis is often seen.

Immunohistochemical expression of neuroendocrine markers synaptophysin and chromogranin A is seen in the majority of PanNETs [76], and peptide hormones (e.g. insulin and glucagon) can also confirm neuroendocrine differentiation. Large cell PanNECs typically express neuroendocrine markers, but small cell PanNECs may not. Both large and small cell PanNECs are typically negative for peptide hormones [77]. Aberrant nuclear TP53 expression is commonly found in PanNECs but is never seen in PanNETs [78]. The main microscopic differential diagnosis consists of acinar cell carcinoma (Fig. 7), pancreatoblastoma, mixed neuroendocrine tumors, and dedifferentiated PDAC (Table 1).

#### Genetic signature: familial PanNET

The vast majority (90 %) of PanNETs occur sporadically, but some occur in the setting of associated familial syndromes including multiple endocrine neoplasia type 1 (MEN1), von Hippel-Lindau syndrome (VHL), neurofibromatosis type 1 (NF1), tuberous sclerosis complex (TSC) and the recently discovered glucagon cell adenomatosis (GCA) [5, 79]. Studies of PanNETs occurring in patients with an underlying genetic predisposition have provided important insight into the genes involved in tumorigenesis of PanNETs. Tumorigenesis in these syndromes follow a hyperplasia-neoplasia sequence in which hyperplastic nodules transform over time into frank neoplasms [79–81]. It is assumed that sporadic cases PanNETs develop through a similar hyperplasia-neoplasia sequence. PanNECs are not associated with germline syndromes and are believed to follow a different pathway of tumorigenesis [78].

#### Genetic signature: sporadic PanNET

Whole exome and targeted sequencing of well differentiated PanNETs (grade 1 and 2) of patients without a familial syndrome showed an average of only 16 nonsynonymous mutations per tumor [82]. Somatic mutations of the *MEN1* gene were found in 45 % of these sporadic PanNETs [82]. Others have previously reported loss of heterozygosity at the *MEN1* locus in 20–45 % of sporadic PanNETs [83, 84]. In addition to prevalent somatic *MEN1* mutations, 45 % of sporadic PanNETs harbored somatic inactivating mutations in *ATRX* or *DAXX*, and 15 % had mutations in mTOR pathway genes (in which TSC1/2 functions) [82]. Remarkably, the alternative lengthening of telomeres (ALT) phenotype, a mechanism of telomerase independent telomere maintenance to overcome cell death, was found to correlated perfectly with loss of *ATRX* and *DAXX* [85–87]. Moreover, many gains and losses have been reported in sporadic PanNETs [88, 89]. *VHL* is deleted in 18 % of sporadic PanNET, and recently allelic loss of *PHLDA3* - a regulator of the mTOR pathway - was found in 70 % of sporadic PanNETs [90, 91].

The genetic alterations in well-differentiated PanNETs (grades 1 and 2) have been compared to those in PanNEC (grade 3). Yachida et al. found that small and large cell PanNECs are genetically similar, but distinct from PanNETs [78]. In PanNECs, activating *KRAS* mutations (2 of 7) and inactivating mutations in *TP53* (4 of 7) and *RB1* (5 of 7) were seen. By contrast, none of these mutations were found in 11 well-differentiated PanNETs. Abnormal expression of the TP53 (95 %) and RB1 (75 %) proteins was also frequently seen in PanNEC, but not in well-differentiated PanNETs. Furthermore, all PanNECs retained expression of ATRX and DAXX, while, as noted above, PanNETs showed loss of expression of ATRX and DAXX in 45 % of cases (Table 4) [78].

**Table 4:** Mutations in pancreatic MEN1 syndrome associated microadenomas and PanNETs, sporadic PanNETs and PanNECs

Neoplasm	Mutations					
	*MEN1*	*ATRX/DAXX*	mTOR pathway	*KRAS*	*TP53*	*RB1*
MEN1 syndrome microadenomas	Up to 100 %	0 %	u	u	u	u
MEN-1 syndrome tumors	Up to 100 %	6 %	u	u	u	u
G1/G2 Pancreatic neuroendocrine tumors	45 %	45 %	15 %	0 %	0 %	0 %
G3 Pancreatic neuroendocrine carcinomas	u	0 %	u	30 %	60 %	70 %

*u* unknown

As mentioned earlier, sporadic PanNETs likely develop through a similar hyperplasia-neoplasia sequence as familial PanNETs. MEN1 syndrome associated PanNETs show loss of the wild-type *MEN1* allele in up to 100 % of cases (compared to 19–44 % in sporadic PanNETs). Loss of the wild-type *MEN1* allele is also seen in microadenomas of MEN1 patients and is therefore an early event [83, 92–94]. Loss of Menin can be demonstrated by immunohistochemistry (Fig. 4c and d). In non-syndromic patients, it is unclear which initiating events cause microadenomas to develop, also bearing in mind that not all sporadic PanNETs have *MEN1* alterations.

*ATRX* and *DAXX* mutations with ALT activation have been reported to correlate significantly with tumor size and T-stage, and are thus considered a late event in tumor progression. In total 45 % of PanNETs have alterations in one of both genes [86, 87]. Although less likely, it is not known if sporadic microadenomas have *ATRX* or *DAXX* alterations.

In contrast to sporadic PanNETs, *ATRX* and *DAXX* alterations were only seen in 6 % of PanNETs from MEN1 syndrome patients (but also as late event) and in 0 % of microadenomas suggesting a less important role for these alterations in MEN1 syndrome tumor progression [95].

#### Epigenetic alterations

Few studies investigated epigenetic alterations in PanNETs. Hypomethylation in *LINE-1* was reported in 20 % of well-differentiated PanNETs, and strongly correlated with poor prognosis and high stage [96]. Other studies found hypermethylation of the tumor suppressor gene *RASSF1A* in 75–80 % of PanNETs with associated decreased protein expression of RASSF1A [97, 98]. Interestingly, the *RASSF1* gene has six other transcriptional variants (B-G), of which RASSF1C was seen to be expressed 10 times higher in PanNET than in normal tissue [98]. An in vitro study found the balance between isoform A and C crucial for the expression of β-catenin, where silencing RASSF1A and expression of RASSF1C promotes the accumulation of β-catenin by inhibiting its hTrCP mediated proteasomal degradation [99], possibly sustaining Wnt signaling in PanNET. RASSF1A furthermore represses miR-21 [100]. Interestingly overexpression of miR-21 which is also upregulated in PDAC, was strongly associated with both a high Ki67 proliferation index and metastasis to the liver [101], potentially giving the RAS pathway a role in higher grades of PanNET [54].

#### MicroRNA

Studies of microRNA expression have suggested that miR-193b is a differential marker for PanNET in tissue and serum compared to normal [102]. MiR-103 and miR-107 were also overexpressed and miR-155 was downregulated in PanNET [101].

#### Changes in gene expression

Analyses of gene-expression patterns in PanNETs have found that a number of genes are upregulated in PanNETs, including oncogenes (e.g. *MLLT10/AF10*), cell adhesion molecules (e.g. fibronectin) and growth factors (e.g. IGFBP3) compared to normal islets. Growth factor IGFBP3 was upregulated significantly more in metastases compared to primary PanNETs. In addition, downregulation of tumor suppressor genes (*NME3*), cell checkpoint proteins (p21/Cip1), and transcription factor JunD that is inhibited by Menin, have been reported [103]. Comparison of gene expression between sporadic PanNETs and VHL associated PanNETs, found that VHL associated tumors follow a specific pathway with upregulation of genes related to hypoxia inducible factor proteins (HIF) and vascular endothelial growth factor (VEGF), both of which regulate angiogenesis [104].

Therapeutically, PanNETs relying on angiogenesis are theoretically targetable by blocking specific pathway components (e.g. VEGF inhibitors) [105–107]. Similarly, PanNETs relying on mTOR activation should be particularly susceptible to everolimus, a drug which has shown to significantly prolong survival [108].

### Solid-pseudopapillary neoplasms

Solid-pseudopapillary neoplasms (SPN) are rare tumors accounting for 1–2 % of all malignant neoplasms of the pancreas. These neoplasms mostly occur in female (90 %) patients at an average age of 29 years (SD: 14). SPNs have a low malignant potential. SPNs are usually limited to the pancreas, but 8 % of patients present with distant metastasis. Disease free survival after curative resection is 95 % [109].

#### Gross and microscopic findings

**Fig. 5:**
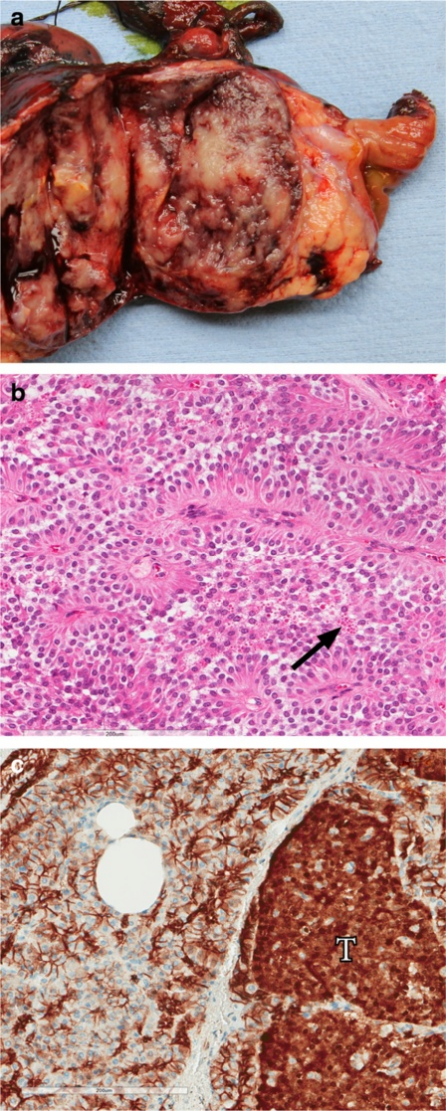
**a** Macroscopic appearance of a solid-pseudopapillary neoplasm showing a well demarcated tumor with solid, pseudopapillary and hemorrhagic-necrotic pseudocystic structures. **b** Microscopically, SPN is characterized by solid areas with relatively uniform cells with eosinophilic or clear vacuolated cytoplasm admixed with delicate capillaries and areas with extensive degenerative changes. The cells are poorly cohesive causing the pseudopapillary appearance. Note the eosinophilic globules (*arrow*). **c** Nuclear β-catenin expression in SPN (*T* tumor) and normal membranous labelling in adjacent normal pancreatic parenchyma

SPNs are essentially solid neoplasms that often undergo dramatic cystic degeneration creating a gross lesion with a mixture of solid, pseudopapillary and hemorrhagic-necrotic areas (Fig. 5a). Microscopically, these neoplasms are composed of poorly cohesive uniform cells clinging ineffectively to delicate capillaries surrounded by extensive degenerative changes. The cells have eosinophilic or clear vacuolated cytoplasm, and the nuclei are round to oval and can be often grooved or indented. Rarely the nuclei are bizarre appearing in areas with degeneration. Eosinophilic globules and foamy macrophages are typically present in these neoplasms (Fig. 5b). SPNs can be distinguished from other pancreatic tumors by the expression of CD10, paranuclear dot-like CD99 labeling and abnormal nuclear labeling for β-catenin (Fig. 5c) or lymphoid enhancer-binding factor 1 (LEF1) [110–114]. The microscopic differential diagnosis consists of neoplasms with a solid and cellular appearance like pancreatic neuroendocrine tumor, acinar cell carcinoma and pancreatoblastoma (Table 1).

#### Genetic signatures: sporadic and familial SPN

Activating mutations in *CTNNB1* (β-catenin) occur in virtually all SPNs, reflected by the nuclear accumulation of β-catenin seen in immunohistochemistry [115, 116]. Recent whole exome sequencing of SPNs found on average of only three non-synonymous mutations per tumor, which is extremely low compared to all of the other pancreatic neoplasms. The *CTNNB1* gene mutations all occur in the critical region between codons 32 and 37 preventing phosphorylation and subsequent degradation of the β-catenin protein.

Two SPNs have been reported in patients with Familial Adenomatous Polyposis (FAP), caused by germline *APC* mutations, confirming that an *APC* mutation is also capable of driving SPN development [117, 118]. The female predominance of SPN is not understood, but it has been shown that estrogenic molecules can influence proliferation in vitro [119].

#### Epigenetic alterations

Undegraded β-catenin in SPNs forms a complex with LEF1, enters the nucleus and activates transcription of several oncogenes including cyclin-D1 that is overexpressed in 70–100 % of SPNs [115, 116, 120]. Cyclin-D1 and its cyclin-dependent kinases phosphorylate the Retinoblastoma (Rb) protein, which drives the cell in the S-phase of the cell cycle. P21 and P27, known to inhibit Rb phosphorylation, were shown to be upregulated in 86 and 100 % of SPNs, respectively. Interestingly, hyperphosphorylated Rb was not detectable, which might explain the low growth-rate of SPN compared to other β-catenin mutated tumors [121].

#### MicroRNA and changes in gene expression

Few studies investigated gene expression and epigenetic alterations in SPN, and all are complicated by the fact that the normal cell that is the counterpart of the neoplastic cell in SPNs has not been identified. These studies are therefore, at best, comparing apples to oranges. One study investigated mRNA and miR expression in 14 SPNs and found 1686 genes to be differentially expressed compared to normal pancreatic parenchyma (which is composed mostly of acinar cells) [122]. These differentially expressed genes activated the Wnt pathway, Hedgehog (HH) pathway, androgen-receptor (AR) pathway and epithelial mesenchymal transition. Moreover, 79 miRs were differentially expressed in these SPNs (49 miRs upregulated, 30 miRs downregulated). By predicting miR targets, 17 of the 30 downregulated miRs possibly upregulated mRNAs in the Wnt/HH/AR pathways [123]. A proteomic profile did not significantly confirm these pathways, but did find upregulation of several proteins involved in the Wnt pathway [122]. Another mRNA analysis in SPN found the NOTCH pathway to be activated in addition to the Wnt Pathway [124]. Large chromosomal rearrangements, aberrant methylation or other non-coding RNAs have not been investigated in SPN.

### Acinar cell carcinoma

Acinar cell carcinoma (ACC) is a rare neoplasm accounting for <1 % of malignant pancreatic tumors. Median age of presentation is 56 years (SD: 15). Most cases occur in late adulthood, but 6 % of cases occur between 8 and 15 years of age. There is no clear syndrome associated with ACC, but ACC has been reported in patients with Peutz-Jeghers syndrome, Lynch syndrome, Familial Adenomatous Polyposis, and in a patient with a germline *BRCA1* mutation [125–128].

Although 60 % of patients with ACC have distant metastasis at presentation (similar to PDAC), overall 5-year survival is 45 % [129]. Some ACCs release digestive enzymes and other products into the blood stream, including alpha-fetoprotein and lipase [130, 131]. About 15 % of patients with ACC present with metastatic fat necrosis, peripheral eosinophilia and arthralgias caused by elevated serum lipase [132].

#### Gross and microscopic findings

**Fig. 6:**
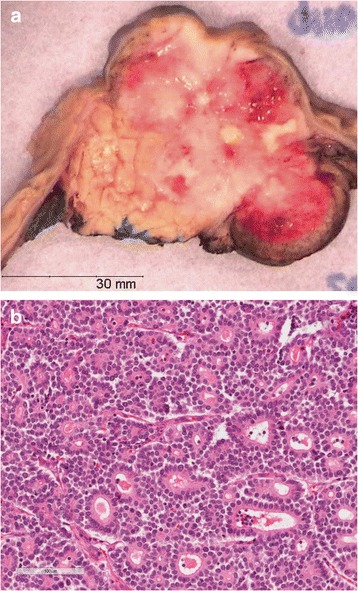
**a** Macroscopic appearance of an acinar cell carcinoma. **b** Microphotograph of an acinar cell carcinoma characterized by a cells with granular cytoplasm and round to oval uniform nuclei forming form small acinar structures

**Fig. 7:**
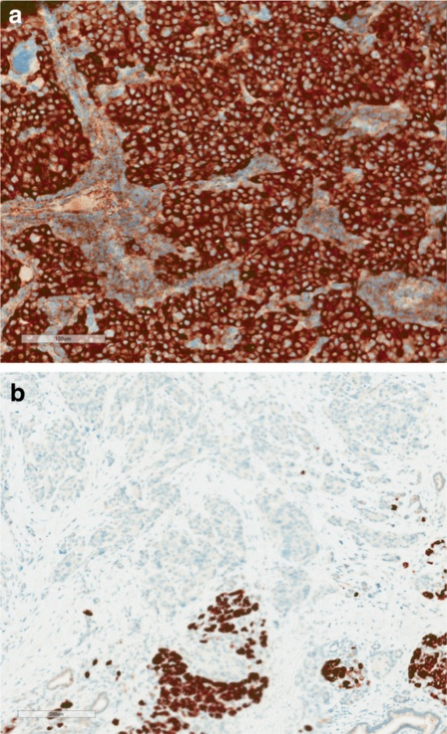
**a** Positive BCL10 expression in an acinar cell carcinoma. **b** Negative BCL10 expression in a pancreatic neuroendocrine tumor

Compared to PDAC, ACCs are relatively soft and well-circumscribed tumors. Microscopically, ACCs are reminiscent of normal exocrine pancreatic cells with enlarged uniform nuclei with prominent nucleoli and finely granular eosinophilic cytoplasm. The cells can form small acinar units or sheets without a distinctive architecture (Fig. 6a and b) [5]. Acinar cell carcinomas express pancreatic exocrine enzymes such as trypsin, chymotrypsin and lipase that can be detectable by immunohistochemistry [132]. BCL10, normally expressed in normal acini, is also expressed in ACC and is helpful in the differential diagnosis between ACC and other pancreatic neoplasms such as PanNET and PDAC (Fig. 7a and b) [133, 134]. Also the monoclonal antibody 2P-1-2-1 can be used to show acinar differentiation [135]. The microscopic differential diagnosis consists of neoplasms with a solid and cellular appearance like pancreatic neuroendocrine tumor, solid pseudopapillary neoplasm, pancreatoblastoma (Table 1).

#### Genetic signature: sporadic ACC

Whole exome sequencing of ACCs revealed that these tumors, on average, harbor a large number of mutations (131 nonsynonymous somatic mutations per tumor in one study). Also, chromosomal instability is seen with a relative high fractional allelic loss compared to PDAC. Chromosome 11p is lost in ~50 % of ACC, suggesting that a locus on 11p may play an important role in ACC development [136, 137]. Many other gains and losses have been reported including loss of the *TP53* locus on 17p (25 %), the *APC* locus on 5q21 (50 %), the *SMAD4* locus on 18q (60 %) and gain of the *CTNNB1* (β-catenin) locus on 3p [137–140]. Whole exome sequencing data further revealed that no single gene was mutated in more than 30 % of ACCs. The genes targeted include *SMAD4* (25 %); *JAK1* (20 %); *BRAF*, *RB1*, *TP53* (13 % each); *APC*, *ARID1A*, *GNAS*, *MLL3*, *PTEN* (9 % each) and *ATM*, *BAP1*, *BRCA2 PALB2*, *MEN1*, *RNF43* (4 % each) [137]. Recently, a review combined all ACC sequencing studies and found similar results: *SMAD4* mutations in 19 % of ACC, *CTNNB1/APC* in 15 %, *TP53* in 12 %, and *BRAF* in 6 % [139].

Ten percent of ACCs appear to be microsatellite instable and may thus be sensitive to immunotherapy [19, 139]. In addition, a number of other potentially actionable mutations, such as *BRCA* and *JAK1* mutations, have been found in ACCs [137]. *BRAF* mutations are rarely seen in ACC; notably, comprehensive genomic profiling identified rearrangements in 23 % of ACC involving either *BRAF* or *RAS*. The most prevalent fusion *SND1-BRAF* activated the MAPK pathway and made the cells sensitive for MEK inhibitor trametinib, so this pathway might be useful as therapeutic target for a subgroup of patients with ACC [141].

#### Epigenetic alterations and MicroRNA

The importance of the *APC/*β-catenin pathway for ACC becomes more evident when methylation is taken into account. *RASSF1* and *APC* were reported to be methylated in 60 and 56 % of ACCs, respectively [142]. A different study confirmed the high percentage of ACC with *APC* methylation, and also found significantly more *MLH1* methylation in ACC compared to PDAC and PanNET [143].

MiR has only been studied in four ACCs in comparison to PanNETs. Surprisingly, 93 % of differentially upregulated miRs and 70 % of differentially downregulated miRs in ACC compared to normal pancreas were also up- or downregulated in PanNET. No specific miR was up- or downregulated in ACC versus PanNET. Overexpression of miR-17, miR-20, miR-21, miR-92-1, miR-103 and miR-107; and lack of expression of miR-155 was found in ACC [101].

### Pancreatoblastoma

Pancreatoblastoma (PB) is another rare tumor with acinar differentiation. PBs usually present in childhood at an average age of 5 years (SD: 2), but there is also a rare group that presents at adult age [144, 145]. The overall 5 year survival is approximately 50 %. PB is associated with Beckwith-Wiedemann syndrome, an (epi)genetic overgrowth-cancer predisposition disorder characterized by exomphalos, macroglossia, and gigantism [146]. As in ACC, serum alpha-fetoprotein and lipase can be elevated in PB and pancreatic panniculitis has also been reported.

#### Gross and microscopic findings

**Fig. 8:**
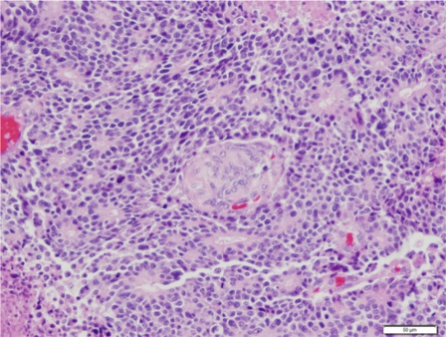
Microphotograph of pancreatoblastoma showing characteristic squamoid nests

Tumors are very similar to ACC in their acinar differentiation. The distinguishing element in PB from other tumors with acinar differentiation are characteristic squamoid nests, which can vary in size and appearance and can even show keratinization (Fig. 8). Neuroendocrine or ductal components may also be encountered, but acinar differentiation and squamoid nests are both required for the diagnosis. PB shares the same immunohistochemical markers for acinar differentiation with ACC, but can also stain positive for markers of ductal or neuroendocrine differentiation. SMAD4 expression is immunohistochemically lost in 20 % [147], and abnormal nuclear expression of β-catenin can be seen, sometimes in the squamoid nests [5, 127]. The microscopic differential diagnosis consists of neoplasms with a solid and cellular appearance like pancreatic neuroendocrine tumor, solid pseudopapillary neoplasm and acinar cell carcinoma (Table 1). In children, other tumors like Wilms tumor and hepatoblastoma should be considered.

#### Genetic signature and epigenetic alterations

Patients with Beckwith-Wiedemann syndrome (germline loss of heterozygosity of chromosome 11p) have a significantly higher risk of pediatric tumors, amongst others pancreatoblastoma which has been reported in several BWS patients [144, 146]. Interestingly loss of 11p also occurs in more than 80 % of sporadic PBs [147]. Likely, several genes on 11p that are expressed according to their parental origin (imprinting) play a role in PB tumorigenesis [148]. The *APC*/β-catenin pathway also plays an important role with 40 to 60 % of sporadic PBs having mutations in *CTNN1B*. In addition, a case with biallelic inactivation of *APC* in a FAP patient has been reported [115, 147]. Aberrant methylation of the promoter *RASSF1A* was seen in in 2 cases [149, 150]. No further characterization in epigenetics has been done.

## Conclusions

The underlying alterations of pancreatic cancer demonstrate that the traditional histopathologic classification of these neoplasms has a solid genetic basis. The genetic changes within each tumor type add to the pathologic classification with the identification of new prognostic markers and new therapeutic targets.

Even with all of the advances in our understanding of genetics of pancreatic neoplasms, the cornerstone to a correct diagnosis is still traditional gross and microscopic examination. Especially the importance of gross inspection is often less appreciated, and yet this can already give important clues to a correct diagnosis. For instance, some tumors are typically solid whereas others are typically cystic. Also, location of a tumor in the pancreas (head, body of tail) and whether a tumor is well circumscribed or ill-defined can point in a certain direction. Most diagnoses of pancreatic tumors can be made without help of additional genetic studies although sometimes proof of a specific genetic alteration in a tumor can further establish a presumed diagnosis. The best example clearly is the SPN, in which virtually all cases have the same underlying *CTNNB1* mutation and immunohistochemistry for β-catenin is routinely used in the diagnostic workup. Also, loss of SMAD4 immunohistochemistry is frequently used in daily practice to suggest pancreatic origin of an adenocarcinoma in a distant site in a patient with a pancreatic mass.

Slowly we are heading towards an era where the combination of classical morphologic pathology and genetic characterization will be essential to establish a more accurate diagnosis. Furthermore, genetic profiling is becoming more and more important for treatment choices; for instance with the choice for a targeted therapy, such as mTOR inhibitors in pancreatic neuroendocrine tumors or PARP inhibitors in BRCA deficient tumors. In the near future, stromal activation, miRNA and methylation markers might influence our choices by better predicting tumor behavior and prognosis. Ideally, would use our knowledge of genetic and epigenetic alterations to screen the blood and pancreatic juices for genetic alteration that identify patients with a high-risk precursor lesion or an early form of cancer. Although our understanding of the genetics of pancreatic cancer has immensely increased in the last decade, many years of research are still needed to integrate all this knowledge and translate it into day-to-day practice.
